# Palatal Mucosal Inflammation Caused by an Unusual Foreign Body in an Infant

**DOI:** 10.3390/diagnostics16030493

**Published:** 2026-02-05

**Authors:** Shunya Ikeda, Yuko Iwamoto, Masashi Ogawa, Tatsuya Akitomo, Ryota Nomura

**Affiliations:** Department of Pediatric Dentistry, Graduate School of Biomedical and Health Sciences, Hiroshima University, Hiroshima 734-8553, Japan; shunyaikeda@hiroshima-u.ac.jp (S.I.); yuko-tulip@hiroshima-u.ac.jp (Y.I.); caries0@hiroshima-u.ac.jp (M.O.); rnomura@hiroshima-u.ac.jp (R.N.)

**Keywords:** foreign body, infant, three-dimensional sticker

## Abstract

Infants may place various objects in their mouths during the developmental process, which can sometimes involve life-threatening risks, such as choking. We describe the case of a 1-year 3-month-old female with a foreign body in the oral cavity. She was referred to our hospital with chief complaints of suspected supernumerary teeth and blisters, and the initial examination revealed blister-like swelling and a white swelling on the hard palate. Intraoral photographs were obtained and examined from multiple angles, revealing findings that resembled a character. Careful re-examination showed that a three-dimensional sticker was attached to the hard palate, which could be removed in one piece. It is important for dental professionals to conduct intraoral examinations of pediatric patients with the understanding that unexpected findings may be present, and think about a foreign body in palatal lesions. In addition, this report highlights a new risk for caregivers supervising infants, as seemingly harmless stickers can remain in the mouth for extended periods.

**Figure 1:**
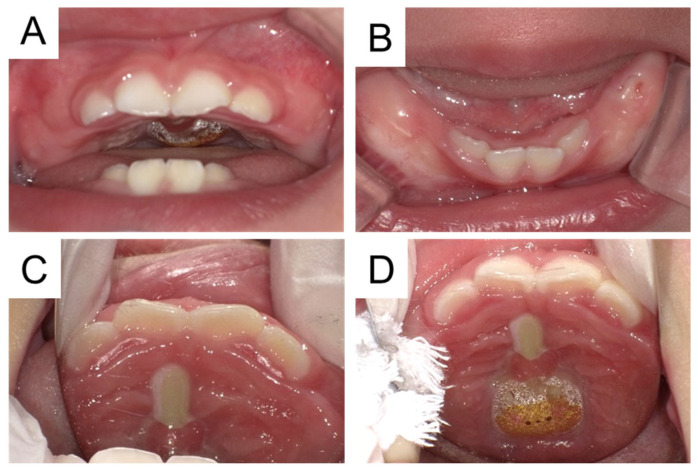
Intraoral photographs at the first visit. A 1-year 3-month-old female presented to our hospital. Her mother had noticed a blister-like lesion in the center of the hard palate and continued observation; however, the condition remained unchanged over time. The patient was referred to our department after visiting a family pediatrician because of suspected supernumerary teeth and blisters. Eight primary incisors and the mandibular left primary first molar had erupted in the oral cavity, and no pathological findings were observed in these teeth (**A**,**B**). A firm, elastic vesicular swelling approximately 10 mm in diameter was detected posterior to the center of the hard palate, and a white swelling measuring approximately 5 mm × 2 mm was detected anteriorly (**C**). There was no spontaneous or contact pain. In addition, the patient consumed a normal diet along with her older siblings and breastfed several times a day, with no noticeable changes in appetite or daily activities after the lesion was discovered. Although atopic dermatitis and wheat allergy were noted, there were no other systemic diseases or relevant family history. When the intraoral photographs were enlarged, a character-like face was observed on the surface of the blister, raising suspicion that a foreign body was adhered to the palate (**D**). By inserting the tip of tweezers into what appeared to be the interface with the surrounding gingiva, part of the foreign body could be lifted and removed in one piece.

**Figure 2:**
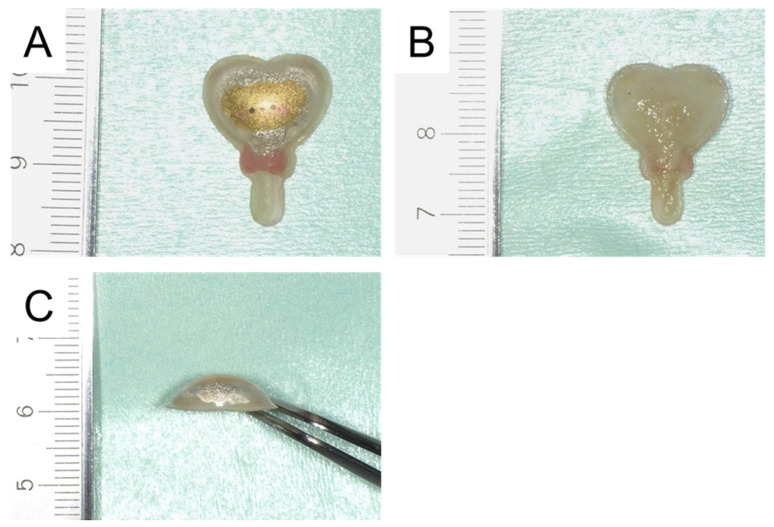
Three-dimensional (3D) sticker removed from the palate. (**A**) The foreign body was a 3D sticker measuring approximately 20 mm × 15 mm, with a heart-shaped upper portion and a narrow, rod-like lower portion. (**B**) The adhesive surface on the back of the 3D sticker appeared swollen, possibly due to moisture absorption. (**C**) The heart-shaped portion was particularly large and thick, measuring up to 4 mm; however, the interior of the sticker was hollow and soft enough to be easily crushed with finger pressure. In the present case, the sticker was tightly adhered with the heart-shaped portion oriented toward the posterior palate.

**Figure 3:**
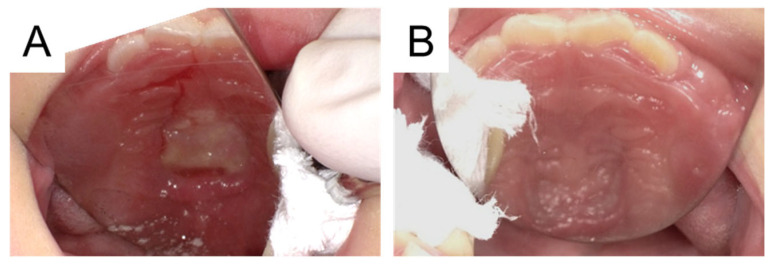
Intraoral photographs after removal of the 3D sticker. (**A**) Following removal, the palatal mucosa exhibited redness and swelling of the surrounding tissues. In addition, the surface that had been in contact with the foreign body had turned white; however, no mucosal damage, such as ulceration, was observed. The area was disinfected with povidone–iodine, and the patient was placed under observation. (**B**) Five days later, re-examination confirmed improvement in the redness and swelling of the palatal mucosa. Infants around the age of 5 months, when chewing functions begin to develop, start placing various objects of interest into their mouths [1]. As calendar age increases, the range of activities expands, making accidents more likely to occur [1]. Ingestion and/or aspiration of foreign body typically affects the pediatric population, mainly children under 4 years of age, with more than 100,000 cases reported annually in the United States [2]. Many of these events are asymptomatic, with swallowed foreign body passing through the digestive tract without causing harm. However, in recent years, the frequency of serious complications caused by ingestion of hazardous items, such as button batteries and magnets, has increased [3]. Additionally, the aspiration of foreign body can rapidly lead to life-threatening airway emergencies, with approximately 300–600 deaths reported annually in children under 15 years of age in the United States [2]. Therefore, the principles of early detection of foreign body and prompt management have been strongly emphasized [2]. In the present case, the 3D sticker entered the patient’s mouth and, fortunately, was neither swallowed nor aspirated, remaining in the oral cavity until the patient’s visit to our department. Impaction of foreign body in the pediatric hard palate is very rare, and to our knowledge, this is the first documented cases of a 3D sticker retained on the hard palate in an infant [4]. In 2019, Khalaf et al. [4] reported a systematic review of foreign body impaction in the pediatric hard palate, in which circular plastic covers were identified in 20 of 32 cases. Although the sticker in this case was not circular, it exhibited a similar morphology, which may predispose such objects to palatal impaction. In addition, impaction in this area is thought to be related to persistent upward forces generated by sucking movements and tongue contact with the palate [4]. In this case, the 3D sticker had a maximum thickness of 4 mm, making it highly likely that the patient initially perceived it as a foreign body in the mouth. However, due to her young age, it was difficult for her to express discomfort. Moreover, its soft structure allowed it to be easily crushed with finger pressure, suggesting that it did not interfere with daily drinking or swallowing, which may explain the absence of changes in food intake. Sustained tongue pressure may also have contributed to the sticker adhering to the palate for a long-term. In the present case, the patient visited their family pediatrician, and the supernumerary teeth and blisters were suspected. Although other differential diagnoses included an eruption cyst, mucocele, and a traumatic lesion, the palate was not a common site, and, in addition, there was no obvious trauma. In recent years, 3D stickers have become popular among children in Japan. However, the patient’s mother had not purchased the sticker responsible for this incident. It had been given to an older sibling by a relative, and the mother was unable to identify a possible cause. Infants may place a wide variety of objects in their mouths; therefore, foreign body should be included in the differential diagnosis of hard palate lesions in children [4]. Approximately three weeks had passed from when her mother first noticed it until the 3D sticker was removed in our department, and it had been there for at least that long. After removal, the attachment surface of palatal turned white, and inflammation was observed in the surrounding tissue. Although it was improved for five days, it was reconfirmed that long-term retention of the foreign body in the palate causes inflammation of the mucosa. This report highlights the potential risks posed by seemingly harmless stickers that can remain in the oral cavity for extended periods. It is important for healthcare professionals to offer preventive guidance about little ornamental items to caregivers during dental and pediatric appointments.

## Data Availability

The original contributions presented in this study are included in the article. Further inquiries can be directed to the corresponding author.
